# Single-molecule sequencing yields the complete chloroplast genome sequence of *Populus deltoides*

**DOI:** 10.1080/23802359.2019.1698346

**Published:** 2019-12-12

**Authors:** Dongyang Wu, Yupeng Wang, Donghua Wu, Lijun Dou, Linming Gao

**Affiliations:** a College of Information Science and Technology, Nanjing Forestry University, Nanjing, Jiangsu, China;; b College of Continuing Education, Nanjing University of Aeronautics and Astronautics, Nanjing, Jiangsu, China;; c Information Center, Nanjing Forestry University, Nanjing, Jiangsu, China;; d Computer Center of Information Science and Technology, Nanjing Forestry University, Nanjing, Jiangsu, China

**Keywords:** *Populus deltoides*, chloroplast genome, phylogeny

## Abstract

*Populus deltoides* is a fast-growing, large tree and one of the largest North American hardwood trees. In this study, the complete chloroplast (cp) genome sequence of *P. deltoides* is characterized. The whole cp genome was assembled to 156,867 bp, including a large single copy (LSC) region of 85,534 bp, a small single copy (SSC) region of 16,513 bp and a pair of inverted repeats (IRs) region of 27,410 bp. The base content of the *P. deltoides* cp genome is A (32.0%), T (31.3%), C (18.0%), and G (18.7%), and AT bases occupy a large proportion of the cp genome. The neighbor-joining phylogenetic analysis with 20 cp genomes from the Salicaceae family showed that *P. deltoides* is sister to *Populus davidiana*. These will provide for the evolutionary and biological studies in Salicaceae family.

*Populus deltoides* (the eastern cottonwood or necklace poplar) is a fast-growing and large cottonwood poplar native to North America. *Populus deltoides* has a large trunk, luxuriant branches, and leaves; it is commonly associated with fast-growth, high-yield, and strong adaptability. It is one of the main tree species for building timber forest, farmland forest network, and four-side tree planting in Shandong, China (Xu and Wang 2012). *Populus deltoides* belongs to the section *Aigeiros* in the genus *Populus*, which has been classified by Eckenwalder (1996). In a plant cell, chloroplast (cp) plays an important role in forming the powerhouse of the cell (Wang et al. 2018). In this study, we reported the complete cp genome sequence of *P. deltoides* and the fully assembled cp genome was submitted to the GenBank database (accession number: MN640592).

Total DNA was extracted from the fresh leaves of *P. deltoides*, which were collected from Chenwei Forest Farm, Sihong County (China; Coordinates: 33°16′7.824″N, 118°21′50.4828″E). The voucher specimen was deposited in the Key Laboratory of Forest Genetics and Biotechnology, Ministry of Education, Nanjing Forestry University (HY01901). Approximately 35.0 GB of raw data were generated by PacBio RS II Sequencing Instrument (Pacific Bioscience, Menlo Park, CA). After quality control and error correction, the clean reads were used to assemble the complete cp genome by Falcon (Chin et al. 2016). Finally, genome annotation was performed with the online program DOGMA (Wyman et al. 2004).

The complete cp genome of *P. deltoides* is 156,867 bp in length, with a typical quadripartite structure of a large single copy (LSC) region of 85,534 bp and a small single copy (SSC) region of 16,513 bp separated by a pair of inverted repeats (IRs) region of 27,410 bp. The overall GC content was 36.7% (LSC, 34.4%; SSC, 30.6%; IRs, 42.1%), similar to other reported species of *Populus*. There are 130 genes, including 85 protein-coding genes, 37 tRNA genes, and 8 rRNA genes. After removing 18 repetitions, protein-coding, tRNA, and rRNA are 77, 31, and 4, respectively. Nineteen genes have introns, three (*clpP*, *rps12*, and *ycf3*) of them have two introns. It is noteworthy that *rps12* is a trans-splicing gene, and its 5′ end is located in LSC region and its 3′ end has one copy in each of the two IR regions.

For a better understanding of the relationships of *P. deltoides* in Salicaceae, the neighbor-joining phylogenetic tree of 15 *Populus* and 5 *Salix* cp genomes based on amino acid sequences of 74 protein-encoding genes (*accD*; *atp-A*, *B*, *E*, *F*, *H*, *I*; *ccsA*; *cemA*; *clpP*; *matK*; *ndh-A*, *B*, *C*, *D*, *E*, *F*, *G*, *H*, *I*, *J*, *K*; *pet-A*, *B*, *D*, *G*, *L*, *N*; *psa-A*, *B*, *C*, *I*, *J*; *pab-A*, *B*, *C*, *D*, *F*, *H*, *I*, *J*, *K*, *L*, *M*, *N*, *T*; *rbcL*; *rpl-2*, *14*, *16*, *20*, *22*, *23*, *33*, *36*; *rpo-A*, *B*, *C1*, *C2*; *rps-1*, *2*, *3*, *4*, *7*, *8*, *11*, *12*, *14*, *15*, *18*, *19*; *ycf1*, *ycf2*, *ycf3*) was constructed by MEGA7 (Kumar et al. 2016; Yu et al. 2018). These protein-encoding genes were selected by an in-house Perl script (Wang et al. 2019). The sister relationship between *P. deltoides* and *Populus davidiana* is strongly supported (Figure 1).

**Figure 1.**
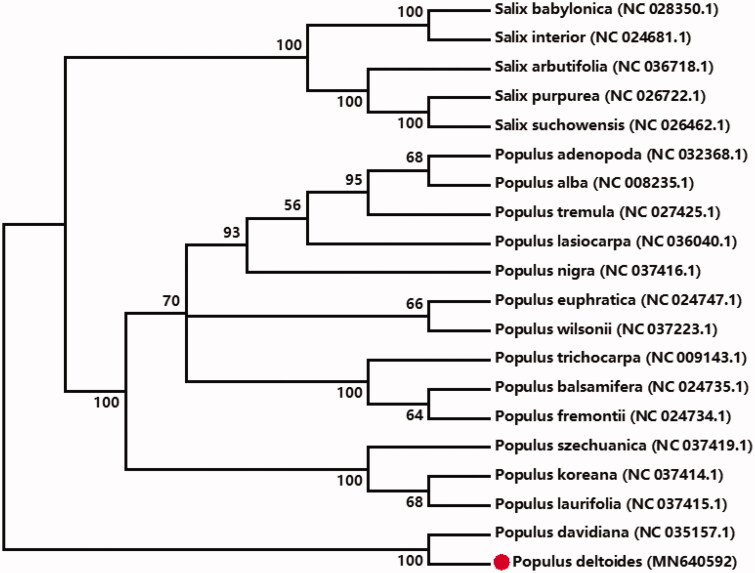
Neighbor-joining method of 20 Salicaceae cp genomes (*Populus adenopoda, Populus tremula, Populus alba, Populus lasiocarpa, Populus nigra, Populus euphratica, Populus balsamifera, Populus trichocarpa, Populus fremontii, Populus davidiana, Populus deltoides, Populus wilsonii, Populus laurifolia, Populus koreana, Populus szechuanica; Salix babylonica, Salix interior, Salix arbutifolia, Salix purpurea, Salix suchowensis*) based on 74 protein-coding genes. The bootstrap values from 2000 replicates are listed for each node.
